# The Recognition of Mental Illness, Schizophrenia Identification, and Help-Seeking from Friends in Late Adolescence

**DOI:** 10.1371/journal.pone.0151298

**Published:** 2016-03-11

**Authors:** Syudo Yamasaki, Shuntaro Ando, Shinji Shimodera, Kaori Endo, Yuji Okazaki, Nozomu Asukai, Satoshi Usami, Atsushi Nishida, Tsukasa Sasaki

**Affiliations:** 1 Department of Psychiatry and Behavioral Science, Tokyo Metropolitan Institute of Medical Science, Tokyo, Japan; 2 Department of Neuropsychiatry, Graduate School of Medicine, The University of Tokyo, Tokyo, Japan; 3 Department of Neuropsychiatry, Kochi Medical School, Kochi University, Kochi, Japan; 4 Kouseikai Michinoo Hospital, Nagasaki, Japan; 5 Tokyo Metropolitan Matsuzawa Hospital, Tokyo, Japan; 6 Aoyamakai Aoki Hospital, Tokyo, Japan; 7 Department of Psychology, University of Tsukuba, Ibaraki, Japan; 8 Department of Physical and Health Education, Graduate School of Education, The University of Tokyo, Tokyo, Japan; Maastricht University, NETHERLANDS

## Abstract

**Objective:**

The recognition of mental illness without anticipating stigma might encourage adolescents’ help-seeking behavior. We aimed to identify the relationship between mental illness identification and adolescents’ intention to seek help if faced with mental illness.

**Method:**

We examined the relationships between help-seeking intentions and recognition of mental illness (RMI) without correctly identifying the disease name, as well as correct labelling of schizophrenia (LSC) using a vignette about a person with schizophrenia in a cross-sectional survey of 9,484 Japanese high-school students aged 15–18 years.

**Results:**

When compared with adolescents who were unable to recognize the mental illness (UMI) in the vignette, those in the RMI group reported they were significantly more likely to seek help from friends (odds ratio [OR] = 1.29; 95% confidence interval [CI] = 1.17–1.41; *P* < 0.001) and expressed an increased likelihood to seek help from professionals (all *P* < .05). Those in the LSC group reported they were significantly less likely to exhibit help-seeking behavior (OR = 0.77, 95% CI = 0.65–0.92, *P* = 0.003) and expressed an increased likelihood of help-seeking from health professionals than the UMI group (all *P* < .05).

**Conclusion:**

The ability to recognize mental illness without identifying the disease may increase help-seeking from friends, while the ability to identify the disease as schizophrenia might decrease late adolescents’ help-seeking. To promote help-seeking behavior among adolescents, improving their ability to recognize mental illness generally is recommended.

## Introduction

Having supportive friends is crucial to helping young people attain optimal physical and mental health in the transition to adulthood [1]. Friends are one of the most important sources of help for mental health problems in late adolescence. Many late adolescents are likely to confide in their friends than in others [2], to disclose their mental health issues only to their friends [3], and to seek help from friends before seeking help from professionals [4–6].

An essential first step to improving help-seeking behavior in young people is promoting awareness of mental illness and education about how to recognize the illness [7]. The ability to recognize mental illness without determining a specific diagnosis has been addressed as one of the key messages in mental health first-aid programs for adolescents [8]. A recent population-based survey found that having the knowledge to accurately identify a patient with schizophrenia from a case vignette significantly decreased help seeking from friends and increased help seeking from medical professionals (e.g. psychiatrist, psychologist, general practitioner, counsellor), while it was not associated with help seeking from family members among young people aged 12–25 years [9]. Having detailed knowledge of schizophrenia might be a barrier to help seeking from friends because of the fear that it would trigger a perception of dangerousness [10], a negative stereotype of the mental illness [11] and evoke anticipated stigma [12]. Educating adolescents on the details of schizophrenia in order to enable accurate diagnostic labelling might be ineffective for promoting help seeking among late adolescents who are likely to seek help from friends.

The recognition of mental illness without anticipating stigma might be a crucial point for encouraging late adolescents to seek help from friends. However, to the best of our knowledge, no epidemiological studies have examined whether the recognition of mental illness is associated with help seeking from friends in late adolescence. In addition, previous studies did not examine help seeking from school-related professionals (e.g. teacher, school nurse, school counsellor), even though schools are an important social environment for adolescent health beyond academic achievement [13].

The aim of the present study was to test whether the ability to recognize mental illness without being able to correctly identify schizophrenia is associated with help seeking from friends among late adolescents between 15 and 18 years of age. Help seeking from friends was compared between two groups: 1) late adolescents who recognized the case in the vignette as having mental illness but did not correctly label the illness, and 2) those who correctly labelled the case with a diagnosis of schizophrenia. We also examined responses from a third group who were unable to recognize the mental illness. We hypothesised that help seeking from friends might be increased in the group that recognized mental illness but not in the group that correctly labelled the case as having schizophrenia. We used a large sample of late adolescents to test these hypotheses.

## Methods

### Study design, sample, and survey procedures

We conducted a cross-sectional survey of public high school students (grades 10–12) in Kochi Prefecture (population: 790,000), Japan, from 2008 through 2009. First, we asked the headmasters/mistresses of all 36 public high schools in Kochi Prefecture if they would be interested in participating in the survey. After consulting with the teachers and parents of their school, 28 headmasters/mistresses agreed to participate in our study. The guidelines for distributing and collecting the questionnaires were given to all of the teachers at the participating schools. The teachers explained to the students that participation was voluntary, confidential, and anonymous. After we had obtained written informed consent from parents, the teachers gave the questionnaires to the students along with envelopes in which the questionnaires were to be sealed after completion, and the research team collected the sealed envelopes from each school.

Of the 9,991 students from the participating schools, 256 were absent on the day that the survey was conducted, and 251 did not agree to participate in this study. Therefore, 9,484 agreed to participate. Among participating students, 714 (7.5%) were excluded from the following analyses due to incomplete answers to the questions on recognition of mental illness, diagnostic labelling of the case, and help-seeking intention. Thus, we analysed the data of 8,770 (92.5%) students who completed the questionnaire. Among them, 4,607 (52.5%) were females, and the mean age was 16.6 years (SD = 0.93; range, 15–18 years). There was no significant difference in age distribution between genders (χ^2^(3) = 0.75, *P* = 0.86). This study was planned and conducted in accordance with the Ethical Guideline for Epidemiological Research of Japan, and approved by the ethics committees of the Tokyo Metropolitan Institute of Psychiatry, the Mie University School of Medicine, and the Kochi Medical School before data collection.

### Measures

The questionnaire included items that (a) assessed the recognition of mental illness and the diagnostic labelling of schizophrenia in a case vignette, and (b) inferred help-seeking intentions if the participant had a mental health problem.

#### Recognition of mental illness and diagnostic labelling of a case

We provided a vignette that described a case of schizophrenia that was based on an interviewer-rated questionnaire that was used in a previous study conducted in Australia and Japan [14]. It presented the symptomatic problems of a person with schizophrenia (see below).

*Hanako lives at home with her parents*. *She has had a few temporary jobs since finishing high school*, *but she is now unemployed*. *Over the last 6 months*, *she has stopped seeing her friends and has begun locking herself in her bedroom and refusing to eat with the family*. *Her parents hear her walking about her bedroom at night while they are in bed*. *Even though they know she is alone*, *they have heard her shouting and arguing as if someone else is there*. *When they try to encourage her to do more things*, *she whispers that she won't leave home because she believes someone is spying on her*. *They know she is not taking drugs or drinking alcohol*.

After reading the vignette, the participants were asked to choose a diagnosis that they thought most appropriately described what the person was experiencing from the following list: *The person is not ill*, *Depression*, *Schizophrenia*, *Eating disorder*, *Social phobia*, or *Unsure*. We classified their responses into three categories: unable to recognize mental illness (UMI: *The person is not ill* or *Unsure*), recognition of mental illness but not correctly labelling the illness (RMI: *depression*, *social phobia*, or *eating disorder*), or correctly labelling the case in the vignette with schizophrenia (LSC: *schizophrenia*).

#### Help-seeking intentions for mental health problems

Help-seeking intentions for mental health problems were assessed with the following question: *If you are troubled by mental distress*, *from whom or where would you seek help first*? Participants were asked to choose one or more items from the various sources of help. We used these answers about help seeking from friends, family members, class teachers, school nurses, school counsellors, and mental health clinicians, which were chosen by over 3% of the participants.

### Statistical analysis

The effects of the responses about the case vignette on help-seeking intentions were investigated with a multivariate logistic regression, in which the help-seeking intentions from each of the six sources (friends, family members, class teachers, school nurses, school counsellors, mental health clinicians) were considered a dependent variable. The model also included age and sex as possible confounding factors. The significance level was set at *P* < 0.05.

## Results

### Help-seeking intentions from friends and other sources

The responses to the questions on the case vignette of schizophrenia and help-seeking intentions are presented in Table 1. The percentage of late adolescents who recognized that the case in the vignette had mental illness (RMI + LSC) was 64.3%. However, the LSC group made up only 7% of the participants. Most (over 60%) of the participants stated they would seek help from friends. Almost half of the participants would seek help from family members. Around 5% of the participants would seek help from class teachers and school nurses, and around 3% would seek help from school counsellors and mental health clinicians (Table 1).

**Table 1:** Participants’ demographic characteristics, questionnaire responses, and help-seeking intentions.

*N* = 8,770	
**Demographic characteristics**	
Mean Age in years (SD)	16.6 (0.93)
Female, N (%)	4607 (52.5)
**Responses to case vignette of schizophrenia**	
UMI, N (%)	3127 (35.7)
RMI, N (%)	4992 (56.9)
LSC, N (%)	651 (7.4)
**Sources of help seeking**	
Friends, N (%)	5628 (64.2)
Family members, N (%)	4062 (46.3)
Class teacher, N (%)	469 (5.3)
School nurse, N (%)	434 (4.9)
School counsellor, N (%)	269 (3.1)
Mental health clinician, N (%)	301 (3.4)

Notes. SD = standard deviation; UMI = unable to recognize mental illness: answered *he/she is not ill* or *unsure* about the case vignette of schizophrenia; RMI = recognising mental illness but not correctly labelling the illness: answered *depression*, *social phobia*, or *eating disorder*; LSC = correctly labelling the case with schizophrenia: answered *schizophrenia*.

### Associations between responses and help-seeking intentions

**Fig 1:**
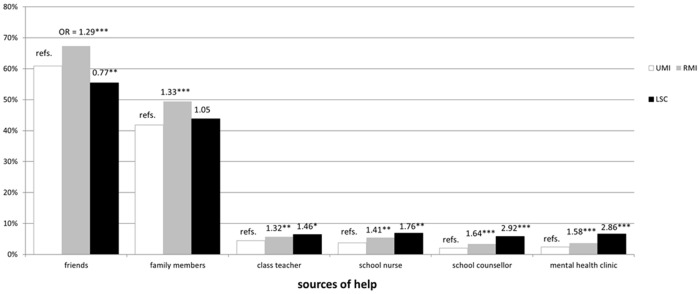
Percentages of subjects in each group seeking help from different sources according to their responses to the case vignette of schizophrenia. Notes. UMI: unable to recognize mental illness; RMI: recognition of mental illness but not being able to correctly label the illness; LSC: correctly labelling the case in the vignette with schizophrenia; OR: odds ratio for the responses to the case vignette of schizophrenia (adjusted for gender and age); refs.: the UMI group was the reference category in the logistic regression. *: *P* < 0.05, **: *P* < 0.01, ***: *P* < 0.001

The percentages of participants with help-seeking intentions in the three groups (UMI, RMI, and LSC) according to the responses to the questions on the case vignette are summarized in Fig 1. The percentage of late adolescents who would seek help from friends was around 70% among those in the RMI group (67.3%), while around half of those in the LSC group (55.5%) and around 60% of those in the UMI group (60.9%) would seek help from friends. Almost half of the RMI group (49.5%) and around 40% of the LSC group (43.8%) and the UMI group (41.8%) would seek help from family. Less than 10% of the adolescents in all three groups would seek help from other professionals (Fig 1).

The results of the logistic regression are summarised in Fig 1. Compared with those in the UMI group, late adolescents in the RMI group stated they would seek significantly more help from friends (odds ratio [OR] = 1.29, 95% confidence interval [CI] = 1.17–1.41, *P* < 0.001), while those in the LSC group would seek significantly less help from friends (OR = 0.77, 95% CI = 0.65–0.92, *P* = 0.003) after adjustment. Those in the RMI group would seek significantly more help from all other sources (family members: OR = 1.33, 95% CI = 1.22–1.46, *P* < 0.001; class teachers: OR = 1.32, 95% CI = 1.07–1.62, *P* = 0.009; school nurses: OR = 1.41, 95% CI = 1.12–1.76, *P* = 0.003; school counsellors: OR = 1.64, 95% CI = 1.22–2.20, *P* < 0.001; mental health clinics: OR = 1.58, 95% CI = 1.20–2.08, *P* < 0.001). Those in the LSC group would seek significantly more help from class teachers (OR = 1.46, 95% CI = 1.02–2.08, *P* = 0.038), school nurses (OR = 1.76, 95% CI = 1.23–2.52, *P* = 0.002), school counsellors (OR = 2.92, 95% CI = 1.93–4.41, *P* < 0.001), and mental health clinics (OR = 2.86, 95% CI = 1.94–4.21, *P* < 0.001), but not family members (OR = 1.05, 95% CI = 0.89–1.25, *P* = 0.567).

## Discussion

In the present study, we found that late adolescents who recognized that the case in the vignette had mental illness but did not correctly label the illness stated they would seek help from friends more than those who did not recognize that the case had a mental illness. In contrast, the percentage of adolescents who would seek help from friends was significantly decreased in the group of late adolescents who correctly labelled the case with schizophrenia.

More than half of the late adolescents would seek help from friends in the present study. These results were consistent with those of a previous study that demonstrated that friends were the most frequent sources of help for mental health problems among late adolescents who were 15–16 years old, while most early adolescents 13–14 years old would seek help from family members [15]. In late adolescence, friends are likely to be the first and most important step when seeking help for mental health problems.

The late adolescents in the RMI group would seek more help from friends, as well as all other sources of help, than those in the UMI group. In contrast, those in the LSC group would seek significantly less help from friends. These findings support our hypothesis and are consistent with the results of previous studies [9, 16]. These findings suggest that educating late adolescents on accurately identifying schizophrenia might be ineffective for promoting help seeking from friends, who are the most familiar and the first step in seeking help for mental health problems in late adolescence.

At the same time, the late adolescents in the LSC group would first seek help two times more often from professionals who were not familiar to them. This is also consistent with previous findings, but the effects were slightly smaller than what was previously found in young people aged 12–25 years old [9]. This effect might be weaker particularly in late adolescence than in the other age group. One possible explanation is that late adolescents who could correctly identify schizophrenia might have more help-seeking intention from friends and less help-seeking intention from health professionals than young people in the other age group. However, we need further evidence to clarify the age-specific effect of the labelling of schizophrenia on help-seeking intention.

In order to promote help seeking from friends, which is often the first step of help-seeking in late adolescence, our findings suggest that it is important to enhance young people’s abilities to recognize mental illness even if they cannot label it as schizophrenia in an educational program. Adolescents must be educated on the general warning signs of mental illness, which are important for recognising mental illness in mental health first-aid programmes [8]. Also, we need to be careful to educate about schizophrenia to prevent stigma against person with schizophrenia. We may need the education program which contains not only the knowledge for diagnosing schizophrenia but also how to appropriately deal with the stereotype of schizophrenia to reduce anticipated stigma against schizophrenia. In addition, our findings suggest that professional sources of help which young people can feel familiar with and can directly access alone might be effective for promoting help seeking in late adolescence. Adolescents who can identify schizophrenia were more likely to seek help from professionals rather than from friends. However, in Japan, late adolescents are unable to seek help first from professionals by themselves because their parents have to allow them to access professional help in the Japanese public health system. Thus, we need to enrich the sources of mental health professional help that can be easily and directly accessed by adolescents on their own. It is potentially beneficial that adolescents are able to first seek help from school-related professionals (e.g. school counsellors, school nurses).

We used a large school-based adolescent sample and differentiated between the recognition of mental illness with or without specific labelling of the mental illness as schizophrenia in order to examine the association among the recognition of mental illness, the specific labelling of schizophrenia, and help seeking from friends and other sources. However, our study had the following limitations. First, we adopted a cross-sectional design, and thus, we could not clarify whether education about schizophrenia or the tendencies of adolescents who already have detailed knowledge about schizophrenia affected their help-seeking intentions. Second, we used a questionnaire and multiple-choice questions about the recognition of mental illness and the labelling of schizophrenia. Therefore, we should consider potential measurement error. In particular, from the 'unsure' responses, we could not differentiate whether the participants identified any psychological/mental/emotional problems or not. Also, from the ‘schizophrenia’ responses, we could not find whether the participants could differentiate between early and chronic schizophrenia. Third, we have a limitation to assess detailed background information about participants which could be potential confounders. Further studies would be needed to reveal the background characteristics of the group which was able to recognize schizophrenia from the vignette. We need to perform future longitudinal or intervention studies in order to better clarify the causal relationship among these issues with a detailed assessment.

## References

[1] Viner RM, Ozer EM, Denny S, Marmot M, Resnick M, Fatusi A et al Adolescence and the social determinants of health. Lancet. 2012;379: 1641–1652. 10.1016/S0140-6736(12)60149-4 22538179

[2] Choquet M, Menke H. Suicidal thoughts during early adolescence: prevalence, associated troubles and help-seeking behavior. Acta Psychiatr Scand. 1990;81: 170–7. 2327280 10.1111/j.1600-0447.1990.tb06474.x

[3] Hunt J, Eisenberg D. Mental health problems and help-seeking behavior among college students. J Adolesc Health. 2010;46: 3–10. 10.1016/j.jadohealth.2009.08.008 20123251

[4] Jorm AF, Wright A, Morgan AJ. Where to seek help for a mental disorder? National survey of the beliefs of Australian youth and their parents. Med J Aust. 2007;187: 556–560. 18021042 10.5694/j.1326-5377.2007.tb01415.x

[5] Rickwood DJ, Braithwaite VA. Social-psychological factors affecting help-seeking for emotional problems. Soc Sci Med. 1994;39: 563–572. 7973856 10.1016/0277-9536(94)90099-x

[6] Rickwood DJ, Deane FP, Wilson CJ. When and how do young people seek professional help for mental health problems? Med J Aust. 2007;187: S35–S39. 17908023 10.5694/j.1326-5377.2007.tb01334.x

[7] McGorry PD, Goldstone SD, Parker AG, Rickwood DJ, Hickie IB. Cultures for mental health care of young people: an Australian blueprint for reform. Lancet Psychiatry. 2014;1: 559–568. 10.1016/S2215-0366(14)00082-0 26361315

[8] Ross AM, Hart LM, Jorm AF, Kelly CM, Kitchener BA. Development of key messages for adolescents on providing basic mental health first aid to peers: a Delphi consensus study. Early Interv Psychiatry. 2012;6: 229–238. 10.1111/j.1751-7893.2011.00331.x 22240091

[9] Wright A, Jorm AF, Harris MG, McGorry PD. What’s in a name? Is accurate recognition and labelling of mental disorders by young people associated with better help-seeking and treatment preferences? Soc Psychiatry Psychiatr Epidemiol. 2007;42: 244–250. 17450404 10.1007/s00127-006-0156-x

[10] Read J, Haslam N, Sayce L, Davies E. Prejudice and schizophrenia: a review of the ‘mental illness is an illness like any other’ approach. Acta Psychiatr Scand. 2006;114: 303–18. 17022790 10.1111/j.1600-0447.2006.00824.x

[11] Angermeyer M, Matschinger H. The stigma of mental illness: effects of labelling on public attitudes towards people with mental disorder. Acta Psychiatr Scand. 2003;108: 304–309. 12956832 10.1034/j.1600-0447.2003.00150.x

[12] Jorm AF, Reavley NJ. Depression and stigma: from attitudes to discrimination. Lancet. 2013;381: 10–11. 10.1016/S0140-6736(12)61457-3 23083628

[13] Sawyer SM, Afifi RA, Bearinger LH, Blakemore SJ, Dick B, Ezeh AC et al Adolescence: a foundation for future health. Lancet. 2013;379: 1630–1640. 10.1016/S0140-6736(12)60072-5 22538178

[14] Jorm AF, Nakane Y, Christensen H, Yoshioka K, Griffiths KM, Wata Y. Public beliefs about treatment and outcome of mental disorders: a comparison of Australia and Japan. BMC Med. 2005;3: 12 16004615 10.1186/1741-7015-3-12 PMC1177951

[15] Rickwood D, Deane FP, Wilson CJ, Ciarrochi J. Young people's help-seeking for mental health problems. Adv Mental Health. 2005;4: 218–251.

[16] Wright A, Jorm AF, Mackinnon AJ. Labels used by young people to describe mental disorders: which ones predict effective help-seeking choices? Soc Psychiatry Psychiatr Epidemiol. 2012;47: 917–926. 10.1007/s00127-011-0399-z 21626056

